# Effect of Homogenization on Microstructure Characteristics, Corrosion and Biocompatibility of Mg-Zn-Mn-xCa Alloys

**DOI:** 10.3390/ma11020227

**Published:** 2018-02-01

**Authors:** Yuan Zhang, Jingyuan Li, Huiying Lai, Yuzhao Xu

**Affiliations:** School of Materials Science and Engineering, University of Science and Technology Beijing (USTB), Beijing 100083, China; zy130481@163.com (Y.Z.); jalaihuiying@163.com (H.L.); xuyuzhaoneu@163.com (Y.X.)

**Keywords:** homogenization, phases, corrosion, TEM, biocompatibility

## Abstract

The corrosion behaviors of Mg-2Zn-0.2Mn-xCa (denoted as MZM-xCa alloys) in homogenization state have been investigated by immersion test and electrochemical techniques in a simulated physiological condition. The microstructure features were characterized using scanning electron microscopy (SEM), X-ray diffraction (XRD), transmission electron microscopy (TEM) and electron probe microanalysis (EPMA), and the corrosion mechanism was illustrated using atomic force microscope (AFM), X-ray photoelectron spectroscopy (XPS) and confocal laser scanning microscopy (CLSM). The electrochemical and immersion test verify the MZM-0.38% Ca owns the best corrosion performance with the corrosion rate of 6.27 mm/year. Furthermore, the film layer of MZM-0.38% Ca is more compact and denser than that of others. This improvement could be associated with the combined effects of the suitable content of Zn/Ca dissolving into the α-Mg matrix and the modification of Ca-containing compounds by heat-treatment. However, the morphologies were transformed from uniform corrosion to localized pitting corrosion with Ca further addition. It could be explained that the excessive Ca addition can strengthen the nucleation driving force for the second phase formation, and the large volumes fraction of micro-galvanic present interface sites accelerate the nucleation driving force for corrosion propagation. In addition, in vitro biocompatibility tests also show the MZM-0.38% Ca was safe to bone mesenchymal stem cells (BMSCs) and was promising to be utilized as implant materials.

## 1. Introduction

Recently, magnesium-based alloys and composites have a crucial role in implant materials applications [1,2,3,4,5,6], due to the moderate mechanical properties similar to bone tissue [7,8,9,10,11,12]. In addition, Mg^2+^ is also the second most important cation for special physiological functions, which can activate the body enzymes, participate in the protein synthesis and muscle growth, and maintain the structure stability of nucleic acid [13]. Besides, Mg-based alloys could be gradually dissolved, absorbed, consumed by metabolic reactions, and then excreted with urinary system after the tissues heal [7,13,14]. Moreover, magnesium alloys have no systemic toxicity in human body and possess excellent biocompatibility with cells activity [15]. Thus, Mg and its alloys have been paid more attention to develop as the candidate biomaterials for clinical applications [1,2,6]. However, these Mg alloys were extremely susceptible to corrosion medium when they were exposed to the physiological condition, leading to the loss of mechanical integrity before the tissues have recovered completely [3]. As a result, how to accurately control degradation rate and explore the corrosion mechanism of biodegradable materials in vitro has become an important problem.

Some efforts have been paid to enhance the corrosion resistance and explore the corrosion mechanism of implant materials [16,17,18]. Unfortunately, almost all these Mg-based alloys consist of aluminum, nickel or other rare earth elements [2,6,9,12,16,17,18,19,20,21,22]. The permanent presence of these elements in the physiological environment may induce latent toxicity and gradually cause chronic diseases (Alzheimer’s disease and muscle fiber damage) [13,23,24,25,26,27]. Therefore, it is essential to develop an adequate degradable alloy. Moreover, the degradation behavior of implant materials depends not only on the type and concentrations of alloying elements but also on the microstructure (grain size and phases distribution) features of the alloys with treatment (temperature, plastic working, and surface modification). Among all of the methods, alloying is an effective means not only for enhancing corrosion resistance with microstructure modification but also endowing the degradable materials with moderate mechanical properties by tailoring the phase morphology, distribution, potential, and size of the matrix [28,29,30]. Besides, the correlations of phase characteristics and degradation behaviors of Mg-xZn (x = 2, 3, 4, 5, 6) alloys were also studied in the simulated body fluid [1,31,32,33,34]. Bakhsheshi-Rad et al. [35,36,37] further discussed that the corrosion behavior of Mg-0.5Ca-xZn (x = 0, 0.5, 1, 3, 4, 5, 6, 9) and Mg-2Ca-0.5Mn-xZn (x = 2, 4, 7) were improved with Zn concentration addition, whereas it would produce a reversed effect with the Zn further addition. Cho et al. [38] also demonstrated that the Mn addition was beneficial to improve the degradation performance of Mg-4Zn-0.5Ca alloy, where the dense film layers of MnO and MnO_2_ on the surface can inhibit the chloride ion permeation and control matrix dissolution. In addition, the β-Ca_3_(PO_4_)_2_ addition in Mg-Zn series alloys were further investigated [39,40]. These results showed that adding β-Ca_3_(PO_4_)_2_ could strengthen the mechanical properties and corrosion resistance via hot extrusion and aging treatment. Based on the above, there is very limited research related to the degradation behavior of Mg-2Zn-0.2Mn-xCa alloys with homogenization treatment in the Kokubo solution. Thus far, the mechanism of phase characteristics (surface Volta potential distribution and interface distinction) on the corrosion resistances of Mg-2Zn-0.2Mn-xCa and 3D corrosion profiles in the Kokubo solution have not been discussed, and the data about biocompatibility of investigated alloys are relatively scarce.

Therefore, in this study, the comprehensive microstructure observation of MZM-xCa alloys subjected to homogenization treatment were investigated by TEM, SEM, EPMA, XRD, XPS and AFM, and their in vitro biocompatibility was also examined. Furthermore, the degradation mechanism is further illustrated.

## 2. Experimental Details

### 2.1. Preparation of the Alloys

The Mg-Zn-Mn-xCa (MZM-xCa) alloys were prepared from high-purity Mg (>99.94%), high-purity Zinc (99.99%), Mg-Ca (20% wt %) and Mg-Mn (5% wt %) master alloys. The melts were held at 750 °C for 20 min under the gas mixture of Ar/SF_6_. Then, the melts were poured into a graphite crucible and cooled at ambient temperature. Before casting, the graphite mold was preheated at 250 °C. The composition analysis was determined by ICP-AES (Varian 715-ES), and the results are listed in Table 1. Afterwards, all billets were subjected to homogenization treatment at 380 °C for 24 h and then cooled by water.

### 2.2. Microstructure Characterization

Samples for microstructure observation were polished down to 0.25 μm, and then were etched using 5 g picric acid, 10 mL acetic acid (17.5 mol/L), 10 mL distilled water and 100 mL ethanol. Then, the microstructure characteristics were detected using SEM (Zeiss Auriga, Oberkochen, Germany) and EPMA (JXA-8100, JEOL, Akishima, Japan). The phases characteristics were detected using XRD (SmartLab) (Rigaku Co., Ltd., Tokyo, Japan) and TEM (Tecnai G2) (Thermo Fisher Scientific, Hillsboro, OR, USA). The surface Volta potential distributions and corrosion profile were detected using AFM (MFP 3D Infinity, Oxford Instrument, Abingdon, UK), which was conducted at 25 °C and a relative humidity of 40 ± 5% [41].

### 2.3. Electrochemical Measurement

According to ASTM G3-89 [42], the polarization measurements were measured in the Kokubo solution using an electrochemical workstation (Princeton Versa STAT 3F, AMETEK, Chicago, IL, USA). The standard three-electrode system consists of a saturated calomel electrode as the reference electrode, a platinum plate as the counter electrode and the as-prepared sample with an exposed area of 1 cm^2^ as the working electrode. The composition of Kokubo solution is listed in Table 2. When the open circuit potential (OCP) reaches the steady-state for 30 min, the measurement was conducted with a scan rate of 1 mV/s. The corrosion potentials (E_corr_, V_SCE_) and corrosion current densities (*I*_corr_, mA/cm^2^) were calculated from the polarization curves by the Tafel extrapolation method. The corrosion rate (*P_i_*, mm/year) was related to corrosion current densities, according to the following equation [35]:

(1) $$P_{i}\;=\;22.85\;I_{\mathrm{corr}}$$

### 2.4. Immersion Test

In accordance with ASTM G31-72 [44], the immersion test was conducted in the Kokubo solution at 37 °C for 240 h, and the ratio of specimen surface area to solution volume was 1:30 cm^2^/mL. Prior to immersion tests, all samples were polished down to 0.25 μm and weighted. After the immersion test, the corrosion products were analyzed by SEM, XPS and XRD, respectively. Subsequently, according to the G1-03 (2011), the corrosion products were chemically removed using a solution containing 200 g/L CrO_3_ and 10 g/L AgNO_3_, and then the corrosion morphologies were characterized by confocal laser scanning microscopy (CLSM) (OLS 4000, Olympus, Tokyo, Japan). The corrosion rate (CR) was determined using the following equation:

(2) $$CR=\frac{K\times W}{A\times T\times D}$$

where *K* is a constant (8.76 × 10^4^ for rate unit of millimeter per year), *W* is mass loss (g), *A* is the sample area exposed to solution (cm^2^), *T* is the time of exposed (h) and *D* is the density of materials (g·cm^3^). Each specimen was tested three times under the identical conditions.

### 2.5. In Vitro Biocompatibility Assessment

Human bone marrow mesenchymal stem cells were adopted to evaluate the cytotoxicity of the investigated MZM-xCa alloys. The BMSCs were cultured in Dulbecco’s modified Eagle’s medium (DMEM, Gibco, Waltham, MA USA) supplemented with 10% fetal bovine serum (FBS, Gibco, Waltham, MA USA), 100 units/mL penicillin and 100 units/mL streptomycin in a cell incubator (humidified atmosphere with 5% CO_2_ at 37 °C). The cytotoxicity tests were carried out by indirect contact. Polished samples were washed, dried in air and sterilized via Co60 γ ray radiation.

Extracts were prepared using DMEM serum free medium as the extraction medium with the surface area to extraction medium ratio 1.25 mL/cm^2^ in a humidified atmosphere with 5% CO_2_ at 37 °C for 72 h, according to ISO 10993-5:1999. The supernatant fluid was withdrawn and filtered to prepare the extraction medium, and subsequently preserved at 4 °C before the cytotoxicity test. The control groups involved the use of DMEM medium as negative controls. The BMSCs were seeded in 96-well plates at a density of 5000 cells/well and incubated for 24 h to allow attachment. Then, the medium was replaced with 100 μL extract or DMEM medium, and incubated for 1, 2 and 3 days, respectively. In addition, the relative growth rate (RGR) was calculated for all samples using the Cell Counting Kit-8 (CCK-8, Sigma-Aldrich, St. Louis, MO, USA). After each assay time point, 10 μL CCK-8 solution was added to each well and then it was incubated for 2 h. The optical density (OD) was measured at the wavelength of 450 nm using a microplate reader (iMARK, Bio-Rad, ‎Hercules, CA, USA). The cell viabilities were expressed as relative growth rate (RGR) determined by RGR (%) = {(OD)sample/OD (negative control)} × 100%. All the data were presented as the mean RGR value ± standard deviation. Statistical analysis was conducted to evaluate the difference in cell viability by analysis of variance (ANOVA), where the statistical significance was defined as *p* < 0.05.

## 3. Results and Discussion

### 3.1. Microstructure Characteristics

**Figure 1:**
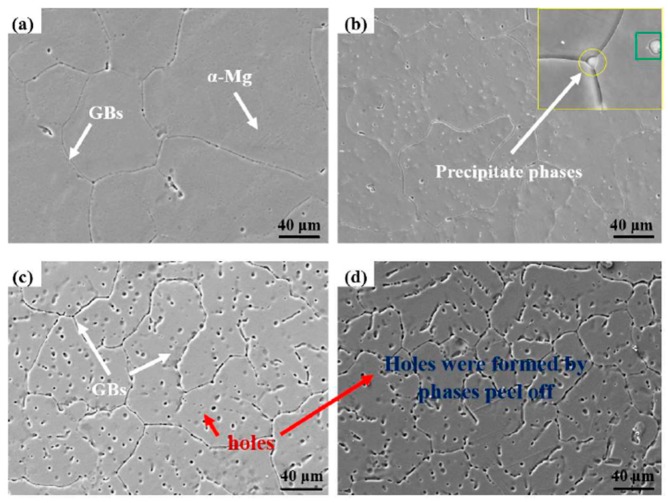
SEM morphologies of as-receive MZM-xCa alloys with homogenization treatment: (**a**) 0%; (**b**) 0.38%; (**c**) 0.76%; and (**d**) 1.10%.

Figure 1 reveals the microstructure evolution of as-homogenized MZM-xCa alloys with various Ca content addition. The grain size within Mg-2Zn-0.2Mn (Figure 1a) alloy was remarkably inhomogeneous, for which the smallest grain size was less than 40–50 μm and the largest size was more than 400 μm. Besides, the MZM has the equiaxed grains, and the average gain size was about 180 μm by the linear intercept methods. The grain size slightly reduced with the 0.38% Ca addition and only a small quantity of precipitation phases were detected in the matrix and triangular grain boundary (the inset of Figure 1b). When the Ca concentration further increased to 0.76% and 1.10%, the grain size of the two alloys significantly decreased and the average grain size was 87 μm and 60 μm, respectively. Some holes were observed in the matrix due to peeling of the precipitates. As for the reason of grain refinement, it was widely accepted that an alloy liquid consisting of a small amount of Ca atoms at the solid/liquid interface could form composition undercooling in the diffusion layer, in which the nucleation particles may be activated in the undercooling region, leading to forming more grain nucleation particles and refining the grain size [35]. Moreover, the Ca atoms, with low speed of diffusion, hindered the grain growth in the interfaces and limited the growth rate of crystal. Thus, the grain size of MZM-xCa alloys clearly refined (Figure 1d).

**Figure 2:**
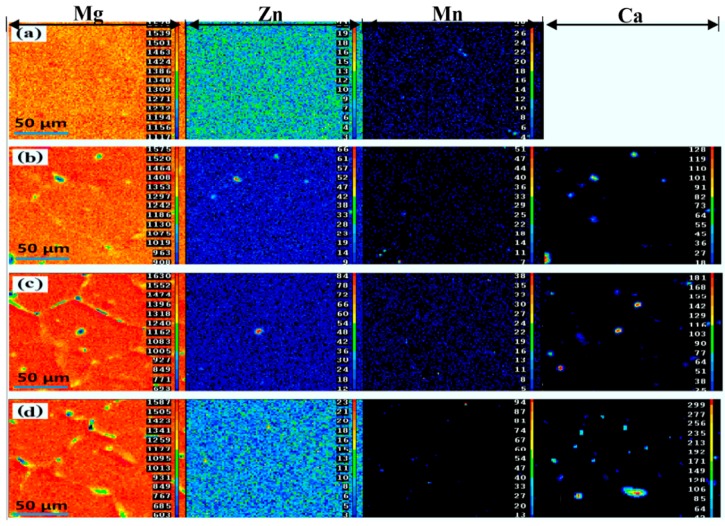
The elements distribution of samples with homogenization treatment: (**a**) MZM; (**b**) MZM-0.38% Ca; (**c**) MZM-0.76% Ca; and (**d**) MZM-1.10% Ca.

**Figure 3:**
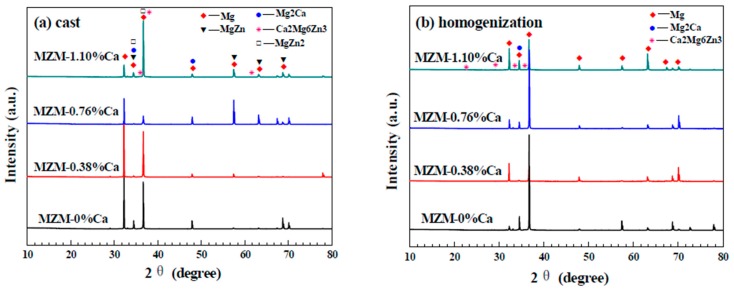

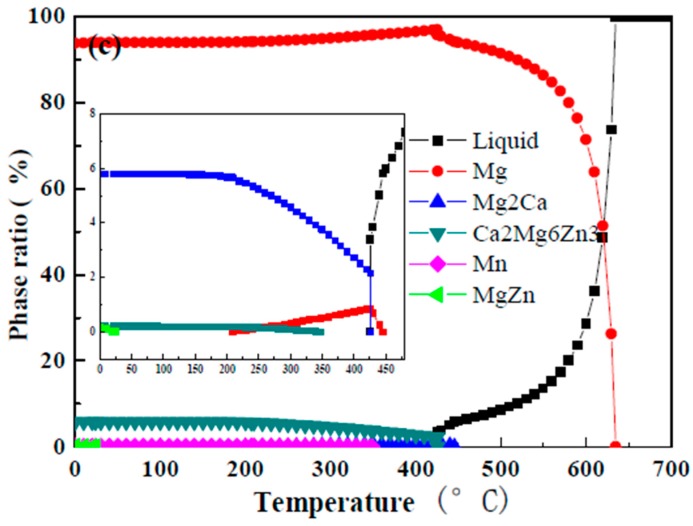
X-ray diffractions patterns of: (**a**) as-cast MZM-xCa alloys; and (**b**) homogenization state; and (**c**) JmatPro calculations results for the microstructure transformation of MZM-1.10% Ca alloy depending on temperature.

Figure 2 reveals the solute element distribution of MZM-xCa with 380 °C/24 h treatment. It can be seen that the distribution of Mn element was uniform along the matrix while Zn/Ca were gathered in localized areas. In addition, the volume fraction of phases (Mg_2_Ca + Ca_2_Mg_6_Zn_3_) increased gradually as the Ca content increased, ranging from 0.26% to 1.93%, which was consistent with SEM morphologies. Figure 3 shows the XRD patterns of studied alloys and compared with as-cast state. It can be concluded that the as-cast MZM-xCa alloys (Figure 3a) were mainly composed of α-Mg, Mg_2_Ca, MgZn, MgZn_2_ and Ca_2_Mg_6_Zn_3_, and the homogenization MZM-xCa alloys (Figure 3b) were composed of α-Mg, Mg_2_Ca and Ca_2_Mg_6_Zn_3_. The reason for the component discrepancy was mainly attributed to Zn/Ca dissolving into Mg substrate after homogenization treatment (Figure 3c).

**Figure 4:**
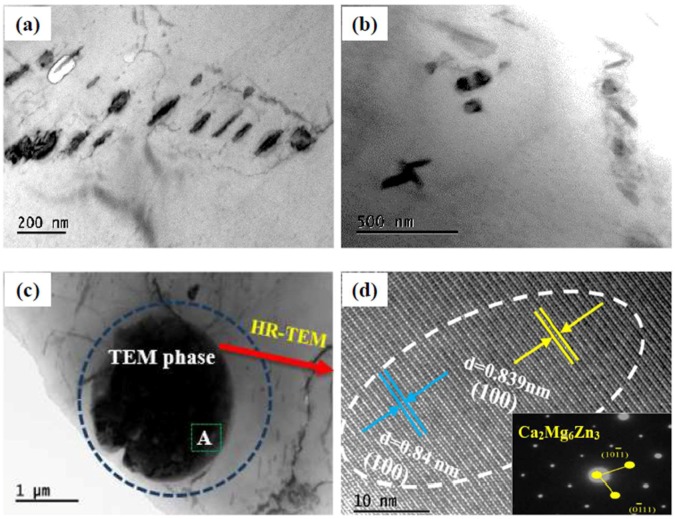

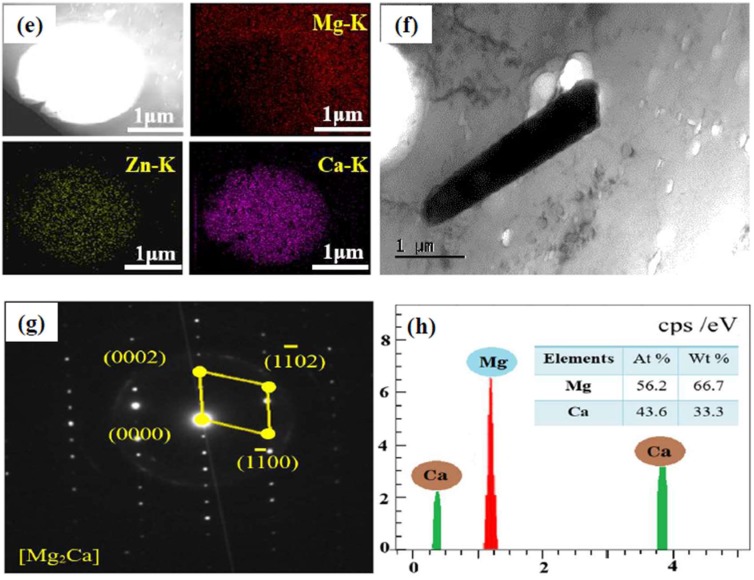
(**a**,**b**) Typical TEM phase features of as-studied MZM-1.10% Ca alloys; (**c**) TEM phase; (**d**) high resolution morphology; and (**e**) element distributions of Ca_2_Mg_6_Zn_3_ marked in (**c**); (**f**) TEM phase; (**g**) selected area diffraction patters; and (**h**) EDS analysis of Mg_2_Ca phase marked in (**f**).

To ascertain the phase structure, TEM observation was further conducted. Figure 4 shows the TEM morphology of MZM-1.10% Ca alloys, and the random distribution of precipitation phases were detected. In addition, Figure 4a–c presents the different morphologies of precipitation phases, such as short-rod shape and granular shape. Figure 4d presents the HRTEM images and selected area diffraction pattern (SADP) of the precipitation (Figure 4c), in which the interplanar space was 0.84 nm. It was consistent with the value of Ca_2_Mg_6_Zn_3_ (100). Moreover, the elemental distribution of precipitates is presented in Figure 4e, which further verified the validity of TEM results. In a similar way, the precipitation of Mg_2_Ca was also identified, and the details are shown in Figure 4f–h.

### 3.2. Electrochemical Measurements

**Figure 5:**
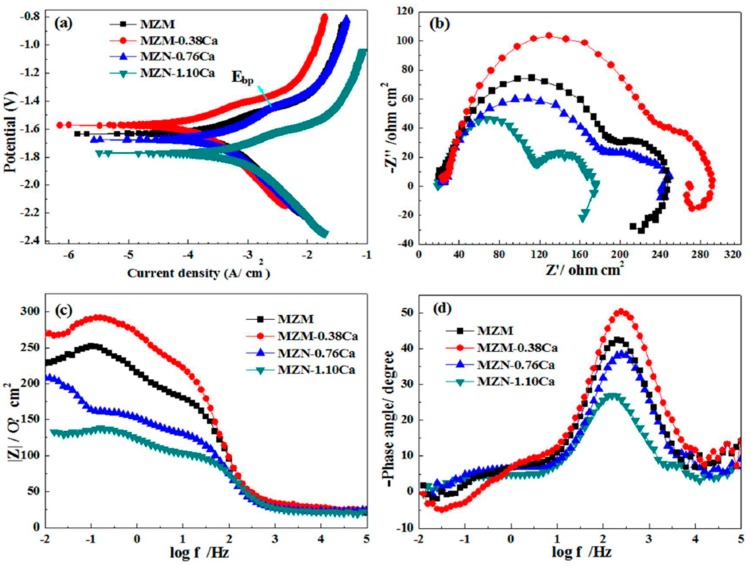
Electrochemical behaviors of MZM-xCa alloys in a simulated body fluid: (**a**) polarization curves; (**b**) nyquist plots; (**c**) bode plots of |Z| vs. frequency; and (**d**) bode plots of phase angle vs. frequency.

Figure 5 shows the electrochemical behaviors of homogenization MZM-x Ca alloys as a function of Ca content in the Kokubo solution at 37 °C, and the fitted results are summarized in Table 3. As indicated, as the Ca content increased, the MZM-0.38% Ca alloys shifted to a more positive potential (−1.57 V_SCE_) compared to the MZM alloy. However, the sample of MZM-(0.76% Ca, 1.10% Ca) unexpectedly shifted to a negative potential (Figure 5a), in which the values were −1.63 V and −1.68 V, respectively. Based on the electrochemical theory, the E_corr_ of samples depends mainly on the relative magnitude between the anodic and cathode reaction rates, which reflected the reaction tendency [27]. Besides, the corrosion current density (*I*_corr_) was presented in a decreasing order: MZM-1.10 Ca < MZM-0.76 Ca < MZM < MZM-0.38 Ca. Lower current density means better corrosion resistance. Thus, it indicated that the MZM-0.38% Ca sample with 380 °C/24 h treatment possesses the noblest corrosion potential, smallest current density and best corrosion resistance. It was associated with the combined effects of the suitable content of Zn/Ca dissolving into the α-Mg matrix and the modification of Ca-containing compounds by heat-treatment. Thus, the corrosion resistance was improved. However, the excess Ca content present in the matrix could generate intermetallic and form micro-galvanic effect, which accelerated the Mg substrates dissolution.

Figure 5b–d reveals the EIS spectra of MZM-xCa alloys in Kokubo solution. In the case of Nyquist plots (Figure 5b), the curve dimensions have different values, which are ranked in an increasing order as follows: MZM-1.10 Ca < MZM-0.76 Ca < MZM < MZM-0.38 Ca, corresponding to an increasing impedance trend of the MZM-xCa alloys in the curves (Bode plots of |Z| vs. frequency) in Figure 5c. As known, the dimension size of the Nyquist plots was the important parameters to reflect the corrosion resistance. Namely, better corrosion behavior of the metal matrix is associated with the higher Z modulus at lower frequency, which is reciprocal to the correlation of corrosion rate for the alloys. In the case of bode phase plot (phase angle vs. frequency), it can be seen that the phase angles of the high frequency capacity resistance crests decrease in the sequence MZM-0.38 Ca > MZM > MZM-0.76 Ca > MZM-1.10 Ca, which can be attributed to the protective performance of the surface film layers. As a result, the test results were consistent with polarization curves.

### 3.3. Immersion Test

**Figure 6:**
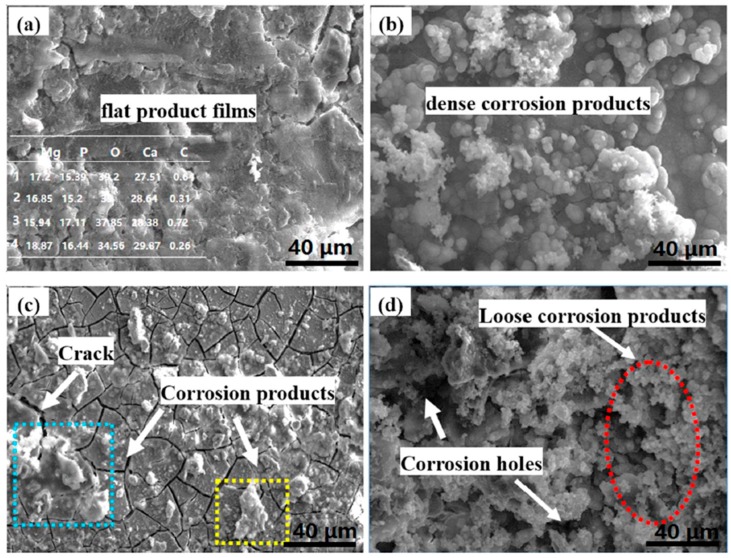
SEM micrographs of corrode surface after immersion test: (**a**) MZM; (**b**) MZM-0.38% Ca; (**c**) MZM-0.76% Ca; and (**d**) MZM-1.10% Ca.

**Figure 7:**
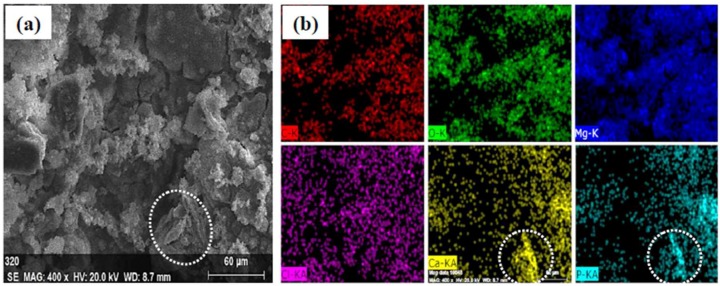
Elements distribution of corrosion products for MZM-1.10% Ca alloys: (**a**) SEM morphologies; and (**b**) main solute elements distribution.

**Figure 8:**
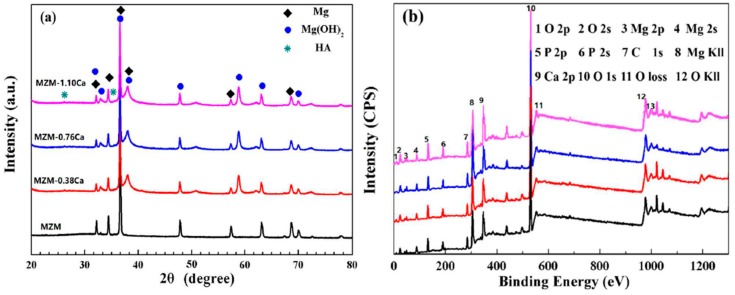
XRD and XPS spectrum of corrosion products of MZM-xCa alloys: (**a**) XRD; and (**b**) XPS.

Figure 6 presents the SEM micrographs of corrosion morphology of MZM-xCa in the Kokubo solution at 37 °C for 10 days. As can be seen, the substantial corrosion products were covered on the surface with cracks features, which was due to the dehydration when they removed from the Kokubo solution. In addition, a flat and smooth surface was obtained in sample of MZM, MZM-0.38 Ca and MZM-0.76 Ca. In contrast, a severe corrosion morphology with large size of pitting holes and multi-defects features was observed in MZM-1.10 Ca. The composition of the products is summarized in the Figure 6 inset. It can be seen that the corrosion products were primarily composed of Mg, O, P, C and Ca elements. Furthermore, the elements distribution of products was shown in Figure 7. These elements such as P, O and Ca corresponded to each other, which indicated the presence of (Mg, Ca)_3_(PO_4_)_2_ (insoluble) in the products. To further study the composition of corrosion products, XRD and XPS were employed to determine the results, as shown in Figure 8. XRD results indicated the corrosion products were primary composed of Mg, Mg(OH)_2_ and a small quantity of hydroxyapatite (HA). As shown in Figure 8b, the XPS test identified the corrosion products consist of O^−^, OH^−^, CO_3_^2−^, PO_3_^2−^, Mg^2+^ and Ca^2+^. Thus, the results of EDS, XPS and XRD have a consistent conclusion, which was also in good agreement with previous studies [6,24,38,45].

**Figure 9:**
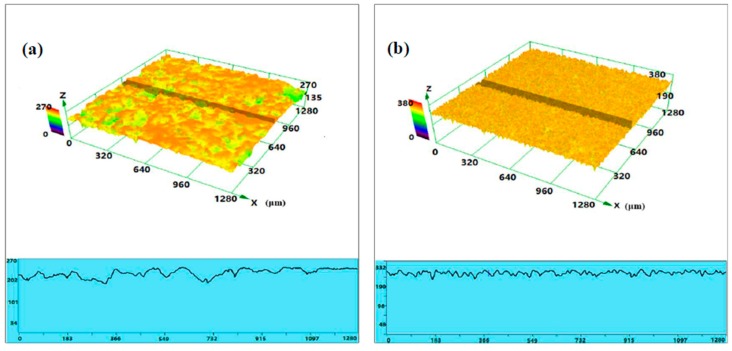

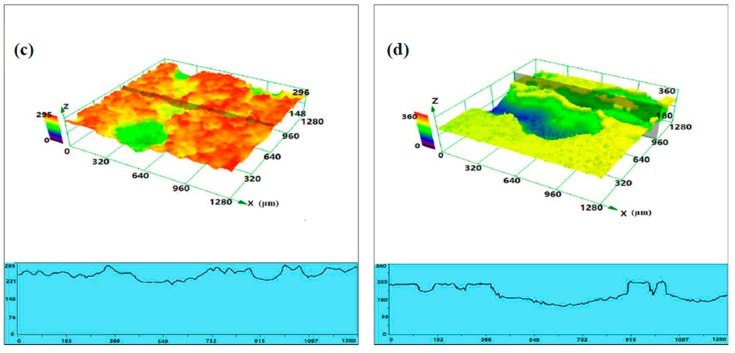
3D corrosion morphology of MZM-xCa alloys with homogenization treatment: (**a**) 0%; (**b**) 0.38%; (**c**) 0.76%; and (**d**) 1.10%.

**Figure 10:**
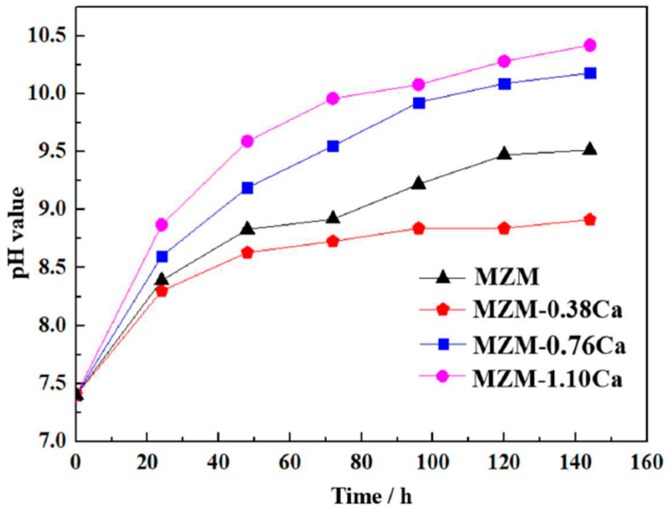
pH value of the studied alloys during the immersion test in Kokubo solution.

Besides, the 3D corrosion morphologies of as-received MZM-xCa alloys after the products removal are shown in Figure 9, which were characterized by laser scanning confocal microscopy (CLSM). As indicated, the experiment alloys surface exhibit the different colors and corrosion profiles, which reflected and represented the different corrosion performance of as-received alloys in a simulated body fluid [45,46,47]. As for the MZM, the surface was slightly corroded and exhibited some shallow pitting holes. The MZM-0.38 Ca alloy revealed a smooth surface, and the fluctuation of profile curves was also relatively slight. With increasing Ca content, the volume fraction of the compounds remarkably increased, which can arise from micro-galvanic corrosion. The large size of pitting holes began to generate and propagate, as shown in Figure 9c,d. Furthermore, the data in Table 4 relate to the corrosion rate *P_i_*. The results from both methods show that addition of 0.38 wt % Ca has the best corrosion resistance. As for the pH measurements (Figure 10), the MZM-0.38 Ca still possesses the low value of pH, indicating the lowest corrosion rate, which was consistent with electrochemical measurements and immersion test.

### 3.4. Biocompatibility Assessment

**Figure 11:**
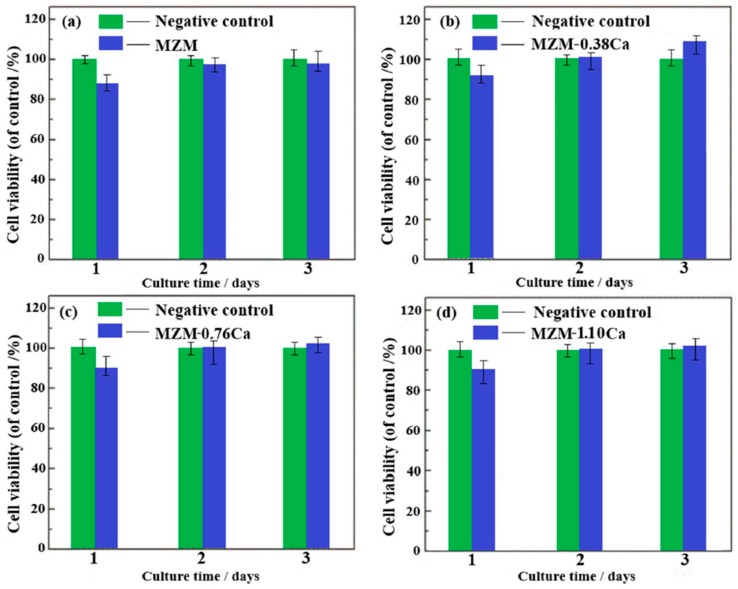
The BMSCs viability in negative control and MZM-xCa alloys extractions after one, two and three days of culture. (**a**) MZM; (**b**) MZM-0.38% Ca; (**c**) MZM-0.76% Ca; and (**d**) MZM-1.10% Ca.

**Figure 12:**
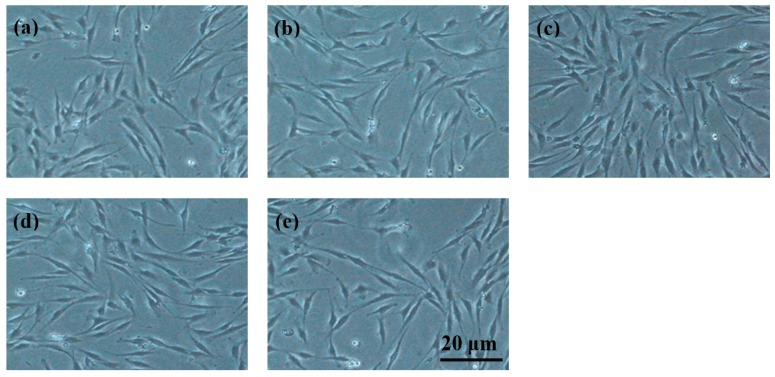
Optical microscopy of BMSCs after three days of incubation: (**a**) negative control; (**b**) MZM extracts; (**c**) MZM-0.38 Ca extracts; (**d**) MZM-0.76 Ca extracts; and (**e**) MZM-1.10 Ca extracts. All the scale bar represents 20 µm.

Cell viability of BMSCs cultured in 100% extraction medium for one, two and three days is shown in Figure 11. It could be seen that, after one day of cultivation, the cells in extracts showed slightly lower viability than that of the control group (Figure 11a). After two and three days in culture of materials’ extracts, the cells from MZM group exhibited still lower viability, but not a significant one, than the negative control group, and the cytotoxicity of these extracts was Grade 0–1 according to ISO 10993-5:1999, indicating that the MZM alloys did not induce toxicity to BMSCs. Nevertheless, as shown in Figure 11b–d, the cells in others extracts exhibited a relatively high viability compared to the former. It indicated that the extracts can offer a suitable environment for BMSCs proliferation. Among the four specimens, the MZM-0.38% Ca alloy exhibits the highest cell viability, reaching 108% after three-day cultivation. The better cell proliferation could be attributed to the synergetic effects of the release of suitable Mg^2+^, Ca^2+^, and Zn^2+^ accompanied by a moderate degradation rate of the MZM-0.38% Ca specimen. In addition, the cells grown in different extracts exhibited a healthy, spindle-shaped morphology, which was similar to that of the negative control group. As a result, the results of cell viability and cell morphology observation (Figure 11 and Figure 12) showed that as-homogenized MZM-xCa alloys did not induce toxicity to cells and met the biocompatibility requirements for implant materials.

**Figure 13:**
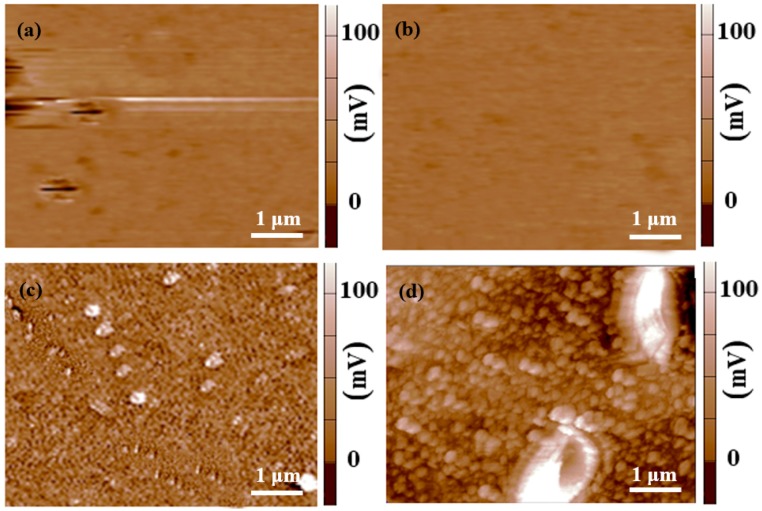
Surface Volta potential distribution of MZM-xCa. (**a**) 0%; (**b**) 0.38%; (**c**) 0.76%; and (**d**) 1.10%.

Aimed to further analyze the corrosion mechanism of MZM-xCa alloys in the Kokubo solution, the SKPFM were used to detect the surface Volta potential distributions. The second phase particles exhibited a brighter color (white color) than the surrounding matrix, indicating the difference in Volta potentials, as shown in Figure 13. In the case of MZM, MZM-0.38 Ca, almost no or trace of precipitatons were detected and exhibited a uniform distribution of Volta potential with relative noble value, corresponding to a better corrosion resistance [45,47]. As for the Figure 13d, the large size of compounds/phases could be observed, which caused the huge difference in Volta potentials between the matrix and phases. Moreover, with the Ca content increased, the large size and volume fractions of phases (Mg_2_Ca and Ca_2_Mg_6_Zn_3_) were observed (Figure 1, Figure 2, and Figure 13), which gathered along the grain boundaries, and exhibited a dots-chains distribution. Furthermore, the morphologies were transformed from uniform corrosion to localized pitting corrosion with Ca further addition (Figure 6 and Figure 9). It could be explained that the excessive Ca addition can strengthen the nucleation driving force for the formation of precipitations (Mg_2_Ca and Ca_2_Mg_6_Zn_3_), and the large volumes fraction of micro-galvanic present interface sites accelerate the nucleation driving force for corrosion propagation.

**Figure 14:**
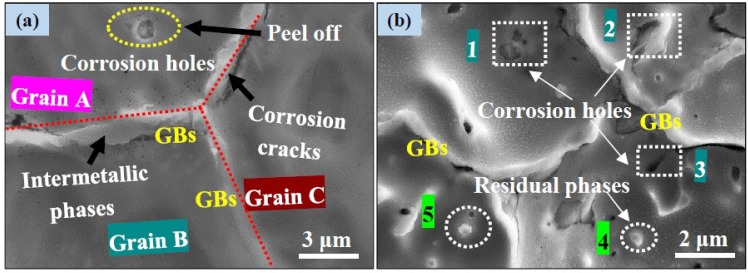
Preferential corrosion areas and morphologies were detected by SEM.

**Figure 15:**
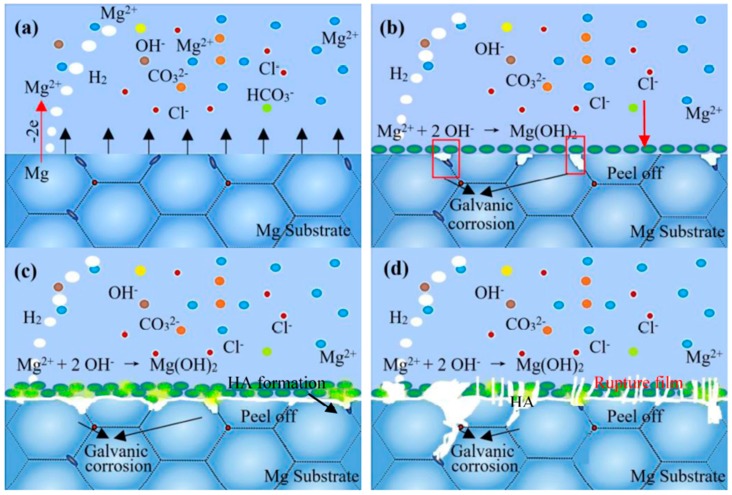
Schematic illustration of corrosion mechanism of as-studied alloys in the Kokubo solution.

Moreover, the preferential corrosion areas were also observed at the interface positions, as shown in Figure 14. The schematic of corrosion mechanism is shown in Figure 15. When the fresh surface exposed to electrode containing corrosive ions, the Mg would immediately transfer into Mg^2+^ and combine with OH^−^ to deposit Mg(OH)_2_ accompanied by the evolution of hydrogen (Figure 15a, Equation (4)). Prolonged the immersion time, the film layer formation and rupture were under a dynamic balance (Figure 15b, Equations (3) and (4)). Besides, the more OH^−^ ions present in the Kokubo solution convert H_2_PO_4_^−^ and HO_4_^2−^ to PO_4_^3−^, and these PO_4_^3−^ ions can react with Ca^2+^ and Mg^2+^ in the electrolyte (Equations (6) and (7)) and result in the formation of calcium phosphate base apatite precipitations according to Equation (8). However, with further extension of the immersion time, the presence of abundant Cl^−^ in Kokubo solution exerts the film layer more active and vulnerable to rupture (Figure 15c, Equation (4)). Besides, the defects, such as micro-cracks and vacancies, can provide diffusion channels for Cl^−^ into film structure to react with MgO and Mg(OH)_2_, which accelerated the dissolution rate of the Mg substrate and caused the formation of localized corrosion (Figure 15d).

(3) $$\left(1-n\right){\;\mathrm{Mg}}^{+}\;+\;\left(1-n\right){\;H}_{2}O\;+\left(1-n\right){\;\mathrm{OH}}^{-}\to \;\left(1-n\right)\;\mathrm{Mg}{\left(\mathrm{OH}\right)}_{2}+1/2\;\left(1+n\right){\;H}_{2}$$

(4) $$\mathrm{Mg}{\left(\mathrm{OH}\right)}_{2}\;+\;2{\mathrm{Cl}}^{-}\;\to {\;\mathrm{MgCl}}_{2}\;+\;2{\mathrm{OH}}^{-}$$

(5) $$H_{2}{\mathrm{PO}}_{4}{}^{-}\;+{\;\mathrm{OH}}^{-}\;\to H_{2}{\mathrm{PO}}_{4}{}^{2-}\;+{\;H}_{2}O$$

(6) $$H_{2}{\mathrm{PO}}_{4}\;+\;2{\mathrm{OH}}^{-}\;\to {\mathrm{PO}}_{4}{}^{3-}-\;+\;2H_{2}O$$

(7) $$3{\mathrm{Ca}}^{2+}+2{\mathrm{PO}}_{4}{}^{3-}\;+{\;\mathrm{mH}}_{2}O\;\to {\;\mathrm{Ca}}_{3}{\left({\mathrm{PO}}_{4}\right)}_{2}\cdot {\mathrm{mH}}_{2}O\left(\mathrm{TCP}\right)$$

(8) $$10{\mathrm{Ca}}^{2+}\;+\;6{\mathrm{HPO}}_{4}{}^{2-}\;+\;8{\mathrm{OH}}^{-}\to {\;\mathrm{Ca}}_{10}{\left({\mathrm{PO}}_{4}\right)}_{6}{\left(\mathrm{OH}\right)}_{2}+\;6H_{2}O\left(\mathrm{HA}\right)$$

## 4. Conclusions

(1) Electrochemical tests and immersion results both showed that MZM-0.38 Ca has the best corrosion resistance, and yields a uniform corrosion morphology on the surface.
(2) The improvement of corrosion resistance could be attributed to the combined effects of the suitable content of Zn/Ca dissolving in α-Mg matrix and the modification of Ca-containing compounds by homogenization treatment.
(3) With the increase of Ca content, nucleation driving forces for the phases formation were facilitated and the microstructure was refined. However, the large volume fraction of micro-galvanic presence between α-Mg and phase accelerates the micro-galvanic corrosion.
(4) In vitro biocompatibility tests show the MZM-xCa alloys were safe to human bone marrow mesenchymal stem cells and were promising to be utilized as implant materials in the future.

## Figures and Tables

**Table 1:** Chemical composition of the experimental alloys (wt %).

Experimental Alloys	Zn	Ca	Mn	Mg
MZM	2.12	/	0.20	Bal.
MZM-0.38 Ca	2.08	0.38	0.20	Bal.
MZM-0.76 Ca	2.11	0.76	0.20	Bal.
MZM-1.10 Ca	2.08	1.10	0.20	Bal.

**Table 2:** Ion concentrations of Blood plasma and simulated body fluid [26,43].

Solution					Concentrations (mM)				Buffer
	Na^+^	K^+^	Mg^2+^	Ca^2+^	Cl^−^	HCO^3−^	HPO_4_^2−^	SO_4_^2−^	
SBF	142	5.0	1.5	2.5	147.8	4.20	1.0	0.5	Tris
Blood Plasma	142	5.0	1.5	2.1–2.6	95–107	27	0.7–1.5	0.5	-

**Table 3:** Result of the electrochemical polarization tests in SBF solution.

Alloys	E_corr_ (V_SCE_)	*I*_corr_ (μA/cm^2^)	*P_i_* (mm/y)
MZM	−1.63	289.93	6.62
MZM-0.38 Ca	−1.576	274.41	6.27
MZM-0.76 Ca	−1.67	356.20	8.14
MZM-1.1 Ca	−1.752	400.31	9.15

**Table 4:** Measurements related to corrosion rate in simulated body fluid at 37 °C.

Alloys	*I*_corr_ (μA/cm^2^)	*P_i_* (mm/y)	E_corr_ (V)	ΔW_m_ (mg/cm^2^/d)	*P_m_* (mm/y)
MZM	289.9	6.61	−1.63	6.95	14.59
MZM-0.38 Ca	274.4	6.27	−1.58	5.62	11.80
MZM-0.76 Ca	356.2	8.14	−1.67	8.87	18.63
MZM-1.1 Ca	400.3	9.14	−1.75	11.18	23.47
